# On the thermal shock resistance and mechanical properties of novel unidirectional UHTCMCs for extreme environments

**DOI:** 10.1038/s41598-018-27328-x

**Published:** 2018-06-14

**Authors:** Luca Zoli, Antonio Vinci, Pietro Galizia, Cesare Melandri, Diletta Sciti

**Affiliations:** CNR-ISTEC, National Research Council of Italy - Institute of Science and Technology for Ceramics, Via Granarolo 64, I-48018 Faenza, Italy

## Abstract

Aerospace provides a strong driving force for technological development. Recently a novel class of composites for harsh environments, based on ultra-high temperature ceramic composites reinforced with continuous fibers (UHTCMC), is being developed. The goal of this work is to overcome the current data patchwork about their microstructural optimization and structural behavior, by showing a consistent mechanical characterization of well-defined and developed UHTCMCs based on ZrB_2_-matrix. The obtained composites have a density of 3.7 g/cm^3^ and porosity of less than 10%. The flexural strength increased from 360 to 550 MPa from room temperature to 1500 °C, showing a non-brittle behaviour. The composites were able to sustain a thermal shock severity as high as 1500 °C. The maximum decrease of strength at 1400 °C was 16% of the initial value, indicating that the samples could be shocked at even higher temperature. Flexural strength, Young’s modulus and coefficient of thermal expansions (CTE) of the composites were measured both along transverse and longitudinal direction and correlated to the microstructural features. The presented microstructural and mechanical characterization well defines the potentiality of the UHTCMCs and can be used as reference for the design and development of novel thermal protection systems and other structural components for harsh environments.

## Introduction

**Figure 1:**
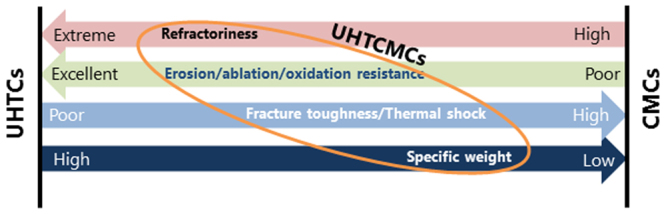
Concept of the new Ultra-High Temperature Ceramic Matrix Composites (UHTCMCs) class, where the arrows point to the desired objective. By wisely combining qualities of CMCs and UHTCs we would like to achieve an increased erosion/ablation resistance compared to pure CMCs, improved damage tolerance and thermal shock compared to UHTCs and as low as possible specific weight.

The pursuit of new ultra-high temperature structural materials is driven by the ever present need to improve the efficiency of aerospace or power-generation gas-turbine engines by operating at higher temperatures. Aeronautical and aerospace applications currently rely on CMC materials based on C/C and C/SiC composite technologies^1^. However, poor resistance to erosion of carbon-based or active oxidation of SiC-based CMCs at temperatures over 1600 °C prevent these materials from being used in even harsher conditions. An alternative class of materials is represented by ultra-high temperature ceramics (UHTCs), such as the borides and carbides of early transition metals^2^. Those materials have been indicated as potential candidates for thermal protection systems but suffer from catastrophic failure due to the lack of flaw tolerance and thermal shock resistance. This paper is focused on a new class of materials which arises from the merging of two distinct classes, one is that of brittle dense UHTCs, one is that of flaw tolerant lightweight CMCs. To overcome present technological limits, novel materials must be conceived, combining the best features of CMCs with those of UHTCs (Fig. 1). Our goal is to design, manufacture and test a new class of out-performing Ultra-High Temperature Ceramic Matrix Composites (UHTCMCs) based on carbon fiber preforms (Cf) enriched with ultra-high temperature ceramics (UHTCs) capable of withstanding operation in severe aerospace environments.

Several issues accompany the research in this novel field of materials: would it be possible to mitigate the limitations of each class of material by merging them? Would it be possible to overcome specific weight issues, poor fiber oxidation resistance and CTE mismatch between the matrix and the fiber with the current compositions? Would it be feasible to fabricate these materials with a time-efficient and green process?

In the literature, the most common approach adopted for the fabrication of UHTCMCs is the introduction of UHTC phase such as ZrC, ZrB_2_, TaC, HfC in C/C or C/SiC composites obtained by PIP or PIP/CVI method^3–5^. The UHTC boride phase is generally added as a paste or a slurry, whilst the carbides can be also introduced using synthesized precursors^6–14^. The amount of UHTC phase is usually lower than the SiC content and the influence of UHTC phase amount on the mechanical and ablation properties has been studied^3^. A. Paul *et al*. have studied the impregnation of C preforms with UHTC-based slurries and subsequent infiltration by CVI. In this case the amount of UHTC phase is predominant in the matrix and SiC (if present) is a secondary phase^10^. In all the aforementioned methods, the UHTC phase is not sintered. Alternative approaches include the reactive metal infiltration process, where a Zr_2_Cu molten alloy reacts with Boron –impregnated Cf preform at temperatures of 1500 °C or higher. The challenge of this process is the complete elimination of unwanted residual low melting phases^15,16^ that even in very low amounts can compromise the high temperature stability.

On the other hand, our process is based on the impregnation of C preforms with UHTC-based slurries followed by sintering via hot pressing or pressure-less sintering^12,14^. One advantage is that the matrix is sintered and thus can contribute to the structural properties and act as thermal barrier for the Cf. The process is remarkably fast and amounts to one working day for the production of the green composite and few hours for sintering.

In this work we present our first prototypes of this new class of materials. The choice of this process is dictated by the need of achieving materials in a timely and green manner: in this respect, long term processes such as CVI or repeated PIP cycles are intentionally avoided. Moreover, the use of toxic chemicals is avoided in any step of the process and aqueous-based, solvent-free processes are preferred. Attention is paid to the easy technology transferability of our process from lab-scale to making sub-scale components. As a baseline material we produced and characterized an unidirectional sample (UD). Our previous work was focused on cross-ply 0–90° architectures, but for a thorough understanding of the thermomechanical behaviour a 0-0° configuration represents the best option to have a baseline material with reference properties. As a matter of fact, the huge variety of solutions adopted in terms of complex preforms, fiber volumetric amount, scattered amounts of UHTC phase in the ceramic matrix, type of coatings and different test methods found in literature make the comparison of results very difficult.

We report the high temperature mechanical testing, including values of bending strength, fracture toughness and, for the first time, thermal shock resistance up to 1500 °C by the water quenching method.

## Results and Discussion

### Microstructural features

Our baseline prototype has the following matrix composition (vol. %): 89% ZrB_2_ + 3% SiC + 8% Si_3_N_4_, where Si_3_N_4_ is added as sintering aid, SiC as reinforcing, oxidantion resistant phase^1,14^. After densification, the composite has a typical fiber content ranging between 40–45%, tailorable through the slurry viscosity, a density around 3.7 g/cm^3^ and porosity between 5–8%.

**Figure 2:**
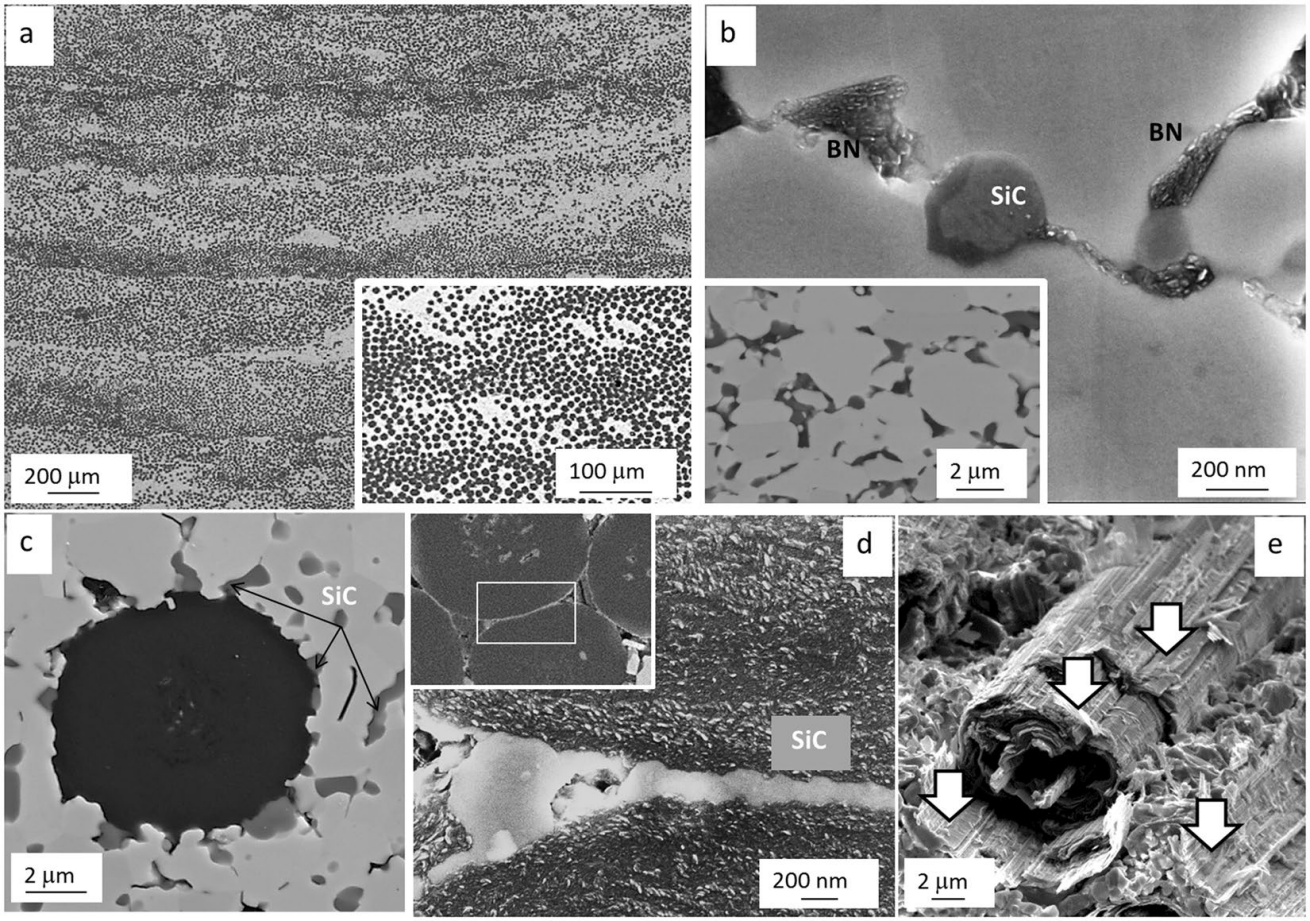
(**a**) Texture of UD samples, (**b**) secondary phases in the matrix, (**c**) example of matrix/fiber interface (BSE imaging), (**d**) SiC layer at the fiber/fiber boundary in the position indicated in the inset, (**e**) detail of fracture surface (arrows highlight different graphitic surfaces produced during the crack propagation).

The typical textures achieved after sintering are reported in Fig. 2. Further details of the microstructure are reported in Fig. 2c–e, showing polished cross matrix features and fiber/matrix interface. Despite the compact microstructure, overlapping of different layers is still visible in the UD samples. As already stated in our previous works, one of the major difficulties in the manufacturing of these composites is dealing with the CTE mismatch between matrix and fiber. ZrB_2_ single crystal has an average CTE of 6.0–7.3•10^−6^ °C^−1^ from RT to 800 °C^17^ and increases up to 7.5•10^−6^ °C^−1^ if ZrB_2_ is produced as polycrystalline ceramic with a certain amount of residual porosity^18^. Pitch-derived carbon fibers have a comparable, although higher, coefficient of thermal expansion in the transverse direction 7.8–8.0•10^−6^ °C^−1^ and a smaller longitudinal coefficient that goes from −1.4 (at RT) to 1.4•10^−6^ °C^−1^ (at 1500 °C)^19^. Micro and macrocracks generated by this mismatch are easily found in the cross-ply 0–90° architectures (not shown here), while they are much less pronounced in the UD sample and occur only along the perpendicular direction of the longitudinal fiber axis. Not only the difference in CTE but also differential shrinkage of matrix and fiber during densification favor the formation of such cracks.

**Figure 3:**
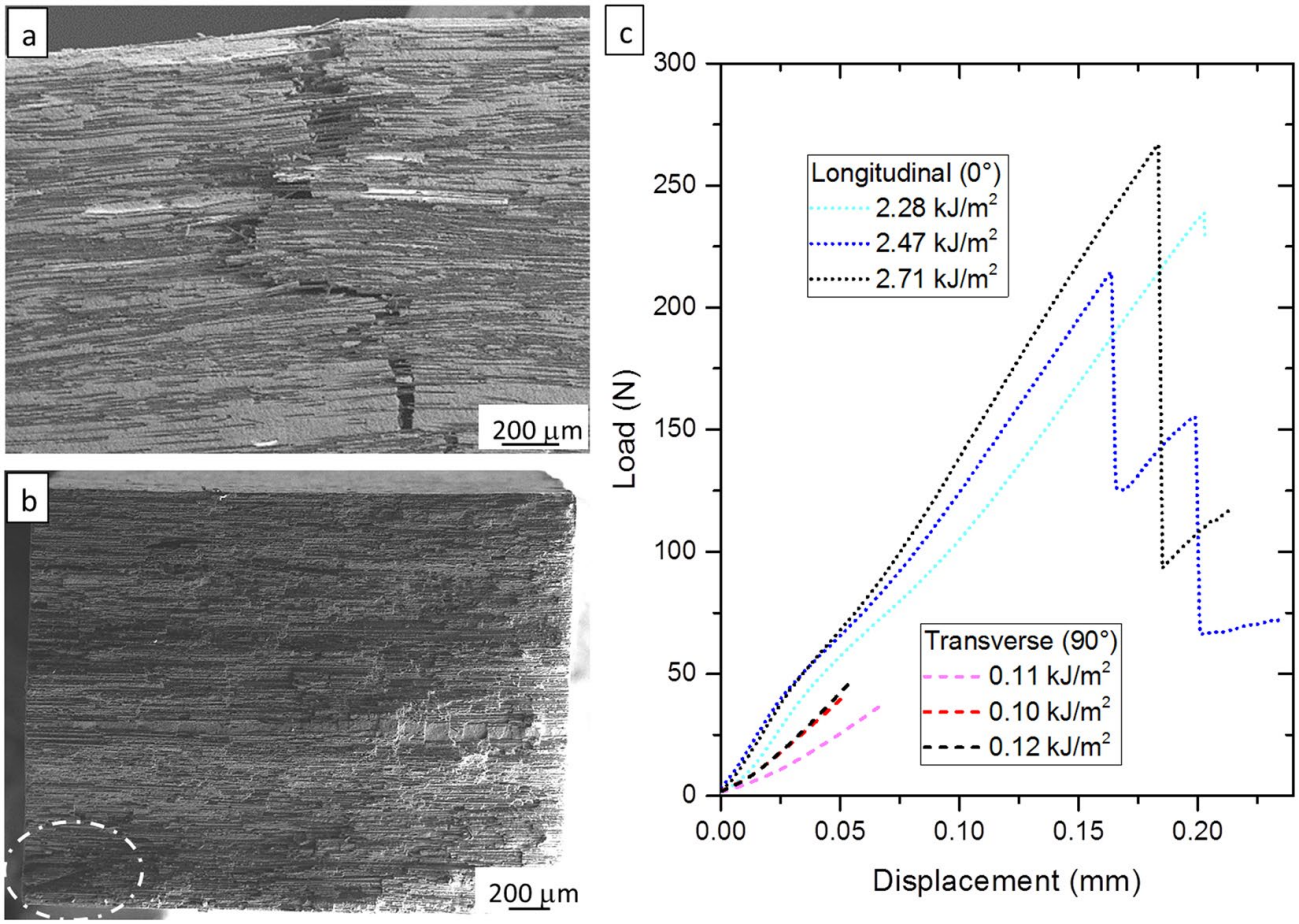
(**a**) Lateral view of failed composite under flexural loading with longitudinal Cf orientation (0°). (**b**) Fracture surface of failed composite under flexural loading with transverse Cf orientation (90°). The possible fracture origin is highlighted by the circle. (**c**) Respective load-displacement curves of 4 point flexural strength for three different bars for each orientation. The adsorbed energy for each bar is reported.

ZrB_2_ grains have dimensions ranging from 2–3 μm and are well sintered. Grey phases represent SiC/SiCN/SiO_2_/BN phases that are evenly distributed in the sintered microstructure, whilst Si_3_N_4_ is no longer detected. The volumetric amount of these low density phases calculated by image analysis is ∼10 vol. % of the matrix. e.g. less than 6 vol. % of the whole composite. Basically, during the densification, Si_3_N_4_ is supposed to react with B_2_O_3_ present as surface oxide of ZrB_2_ to form a Si-O-B-N liquid phases that favors the removal of oxygen bearing species (B_2_O_3_, ZrO_2_)^20^ from the boride particles and their rapid rearrangement. During cooling from the sintering temperature, precipitation of BN phases occurred. An overall reaction explaining these mechanisms is the following:

1 $${{\rm{S}}{\rm{i}}}_{3}{{\rm{N}}}_{4}+2{{\rm{B}}}_{2}{{\rm{O}}}_{3}\to 4{\rm{B}}{\rm{N}}+3{{\rm{S}}{\rm{i}}{\rm{O}}}_{2}$$

favorable at all temperatures. In the case of the present composites, containing a very high amount of C, silica was carbo-thermally reduced to SiC. In Fig. 2b a SiC particle with the original core and re-precipitated SiC-layer is shown. An example of the matrix/fiber interface is illustrated in Fig. 2c–e. Generally speaking, a good adhesion is found between matrix and fiber. SiC particles are preferentially found along the interface. ZrC particles are also found at the interface (not shown) and result from the carbo-thermal reduction of ZrO_2_^21–23^

2 $${{\rm{ZrO}}}_{2}+{\rm{3C}}\to {\rm{ZrC}}+{{\rm{2CO}}}_{(g)}$$

sometimes SiC can be found as a 150 nm thick layer between the fibers, see Fig. 2d. It is presumable that the liquid Si-O based phase flowed between the fibers and was subsequently carbo thermally reduced to SiC. Figure 3a,b illustrates the peculiar way of fracture for the carbon fibers that tend to exfoliate like onion layers^24^. Thus, even if the outer fiber layer is very well adherent to the matrix, fiber pull-out still occur thanks to sliding of the onion-like shells Fig. 2e.

### Thermo-mechanical properties

Table 1 summarizes the results of thermo-mechanical testing. Sample bars for mechanical testing were machined from the sintered pellets by diamond tool machining. Furthermore, since these composites have a sintered matrix, we opted for the standards typical of advanced ceramics rather than those for CMCs. In the literature there is not a standardized approach for mechanical testing of UHTCMCs. The most frequently reported property is 3-pt flexural strength, with span to thickness ratio varying from 8.5 to 12^4–6,16^. In the standards for CMCs span-to-thickness ratio varies from 16 to higher values, indicating that the approach followed so far is closer to the advanced ceramics standards rather than CMCs standards. For our materials the bending strength was determined by 4-pt bending, span to thickness ratio (20 − 10)/2 = 5. The measured values reach 355 MPa at room temperature, improving up to 550 MPa at 1500 °C in Ar flux. Analysis of fracture surfaces shows the fracture surface after bending at 1500 °C, revealing non – brittle fracture and fiber pull-out. Load displacement curves (Fig. 3c) are also shown and the calculated work of fracture (calculated as the area beneath the load-displacement curve divided by the twice the projected real surface) is 2.49 kJ/m^2^. The high energy dissipated during the test is typical of fiber-reinforced composites and is comparable to similar UD ZrB_2_/Cf UHTCMCs^25^, where toughening mechanisms, such as matrix cracking, interfacial debonding and fiber pull-out, are activated (Fig. 3a). The load-displacement curves confirm also that the Cf and the matrix are initially well-bound (as seen from the SEM analysis) so that load is shared and Cf and matrix deform together. In fact, the initial stiffness (which corresponds to 230–240 GPa of Young’s modulus), is 6% higher with respect to the final one, and stiffness reduction occurs when the deformation approaches the failure strain of the composite with transverse configuration. In this case, the strength (65 MPa) seems to be dominated by the matrix properties, and/or the fiber matrix interface strength and the fracture is brittle^26^. The energy dissipated is 0.11 kJ/m^2^. The fracture surface (Fig. 3b) suggests that the fracture origin could be a region where the fibers were not properly infiltrated, as highlighted in the picture. This means that, in the longitudinal configuration, the matrix reaches its own failure stress at about 1/5 of the composite stress levels (65 MPa/355 MPa), and will begin to crack throughout the body of the material. The final bending stress of 355 MPa indicates that, when the matrix fails, the stress on the composite is not enough to break the Cf. Hence the volumetric Cf fraction is higher than the critical value, and the fibers act as effective reinforcement^26^. In other words, the interface between fiber and matrix guarantees the load transfer, and fibers amount is enough to bear the load when the matrix reaches its failure stress. On the other hand, the lower value with respect to the expected one of 600–700 MPa (calculated by the product of the Cf volumetric fraction per Cf tensile strength), and its increase with the test temperature (in both longitudinal and transverse configuration), indicates that the mechanical properties could be influenced by the residual stress arising from the different CTE along the longitudinal direction.

**Table 1:** Thermo-mechanical properties in longitudinal (//) and transverse (⊥) configuration. *Young’s modulus measured using the resonance frequency method.

	Bending strength, σ_f_ (MPa)			Fracture toughness, K_IC_ (MPa•m^1/2^)		CTE (10^−6^ °C^−1^)	Young’s modulus, E (GPa)
	RT	1200 °C	1500 °C	RT	1500 °C	RT-1500 °C	RT
//	355 ± 40	500 ± 51	547 ± 80	9.6 ± 0.7	8.7 ± 1.2	1.96	239 (232 ± 10*)
⊥	63 ± 7	92 ± 13	112 ± 18	—	—	8.30	188

Bending strength values obtained at room temperature are generally higher than those found in the literature for similar composites, even if a direct comparison is still difficult due to different testing conditions, as previously mentioned. H. Hu in ref.^3^ reported a flexural strength of 163 MPa for a C/SiC material enriched with about 25% of ZrB_2_, Q. Li^5^ reported a bending stress of 248 MPa for a 3D-C/SiC enriched with 23% of ZrB_2_-ZrC phases, L. Li^4^ found a value of 255 MPa for 2D-C/SiC enriched with a ZrB_2_-TaC mixture. Another recent contribution by C. Yan *et al*. focused on 3D Cf HfC-SiC composites indicates^6^ reported values of flexural strength ranging from 244 to 392 MPa depending on HfC/SiC ratio. In this case however, the amount of SiC is always higher than the UHTC phase (HfC). A. Paul^27^ found a value of 111 ± 20 MPa for a Cf-HfB_2_ composite determined in 4-pt bending, and span to thickness ratio (80 - 40)/10 = 4. Also in this case direct comparison is not valid since fiber volume fraction, and amount of porosity are not reported. In our previous work^25^, we attested the flexural strength of SiC-free Cf/ZrB_2_ UHTCMC to 284 ± 40 MPa.

**Figure 4:**
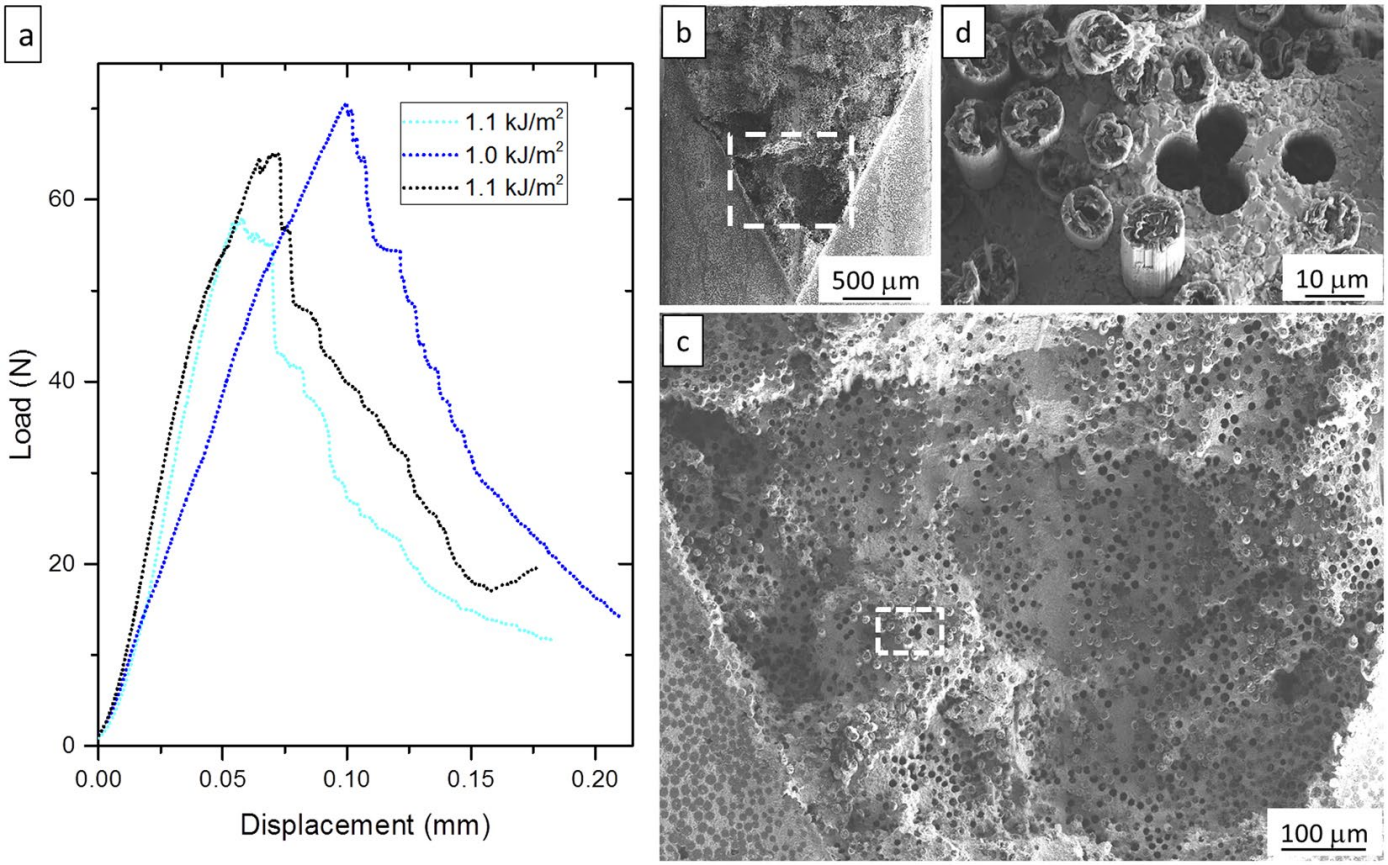
(**a**) Load-displacement curves of 4 pt. for three different chevron notched beams (CNB) with longitudinal orientation. The adsorbed energy for each bar is reported. (**b**) SEM images of fracture surface of failed CNB under flexural loading. (**c**) Magnification of the area highlighted in (**b**), showing holes left by pulled out fibers. (**d**) Magnification of the area highlighted in (**c**), showing length of the pulled out fibers.

The fracture toughness tests also outperformed the values obtained for sintered UHTCs or short fibers reinforced materials, reaching values up to 10 MPa•m^1/2^. Typical load displacement curves at RT are shown in Fig. 4a. Despite the lack of a fiber coating, the obtained toughness approaches the 11 MPa•m^1/2^ reached with SiC-free Cf/ZrB_2_ UHTCMC^25^ where the Cf were distributed as bundles in order to offer substructures able to multiply the toughening mechanisms. As it can be seen from the fracture surface (Fig. 4b–d), for the UD composites the main contribution to the toughness seems to arise from the crack deflection rather than the crack-tip-shielding mechanisms such as the fibers bridging and their pull-out. In fact, although the pull-out is lower than 20 µm, the volumetric shuffling of the fracture surface is pronounced and can be considered a direct consequence of the crack deflections. At 1500 °C the averaged fracture toughness slightly decreases to 9 MPa•m^1/2^. Due to the large data scattering, fracture toughness values obtained at RT and high temperature can be considered comparable within the experimental error. In our opinion the data scattering at 1500 °C could be affected by the growth of the cracks population during the temperature ramp, owing to the different CTE between matrix and Cf.

Values of mechanical strength and fracture toughness at high temperature are hardly reported in the literature for these new composites. Not directly comparable but worthy of note, a C/SiC-ZrB_2_ composite tested at 1800 °C maintained about 74% of its pristine strength. The Cf-HfB_2_ composite produced by A. Paul^24,27^ tested at 1400 °C in flowing Ar, gave a value of 103 ± 25 MPa, which is slightly lower than the value obtained at RT. No data are available for fracture toughness at high temperature.

**Figure 5:**
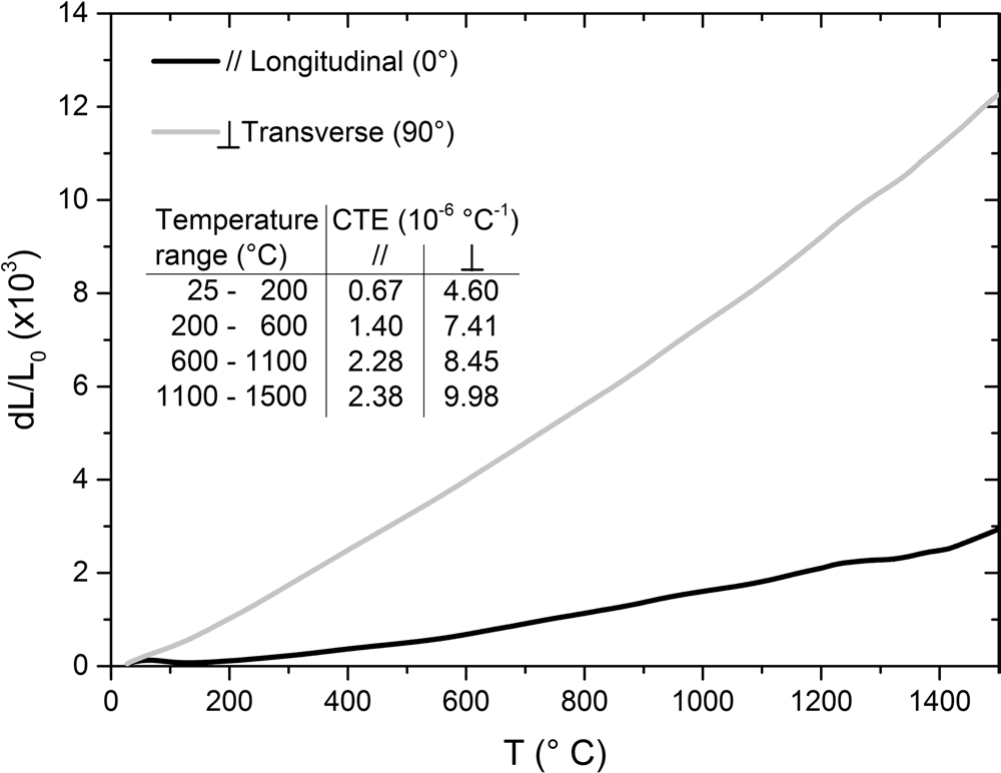
Thermal expansion of UD ZrB_2_/Cf samples measured along transverse (gray line) and longitudinal (black line) orientation plotted as the relative elongation with respect to the original specimen length at room temperature (L_0_). Coefficients for thermal expansion (CTE) averaged over some temperature ranges are indicated in the figure.

The CTE is very different when tested in the direction along the fiber axis or perpendicular to the fiber axis (Fig. 5), which is somewhat expected given the starting CTEs of the constituent phases, and confirms the good mechanical coupling between Cf and matrix. ZrB_2_ containing secondary phases has a CTE variable from of 7.5 to 8.2•10^−6^ °C^−1^, between RT and 1300 °C, according to our previous works^18,28^. As said, the pitch-derived Cf have an anisotropic CTE, which along the transverse direction is comparable with that of the matrix, and lower in the longitudinal direction. In this direction the CTE difference between the Cf and the matrix is 9•10^−6^ °C^−1^ at RT and decrease down to 6•10^−6^ °C^−1^ at 1500 °C. This huge difference should be the reason of (i) the cracks formation in the matrix along the directions perpendicular to the Cf axis, (ii) the matrix failure under low stress 65 MPa (if referred to the 350 MPa of the ZrB_2_ monolithic materials^18^), and (iii) the increasing of the strength with the temperature.

Also for the CTE it is not easy to make comparisons with the few data reported in the literature. For example, although we found a comparable longitudinal CTE with the A. Paul’s Cf-HfB_2_ composites (1.63•10^−6^ °C^−1^)^27^, the transverse CTE is almost doubled (8.30•10^−6^ °C instead of 4.67•10^−6^ °C^−1^ reported in ref.^27^). The different CTE dependence on fiber orientation, which changes by a factor of 4.2 instead 2.9, can be addressed to the different Cf distribution (UD aligned fibers in the present work and arranged in a complex 2.5D structure in ref.^27^) and different type of Cf (pitch-derived in our case, polymer-derived in ref.^27^).

**Figure 6:**
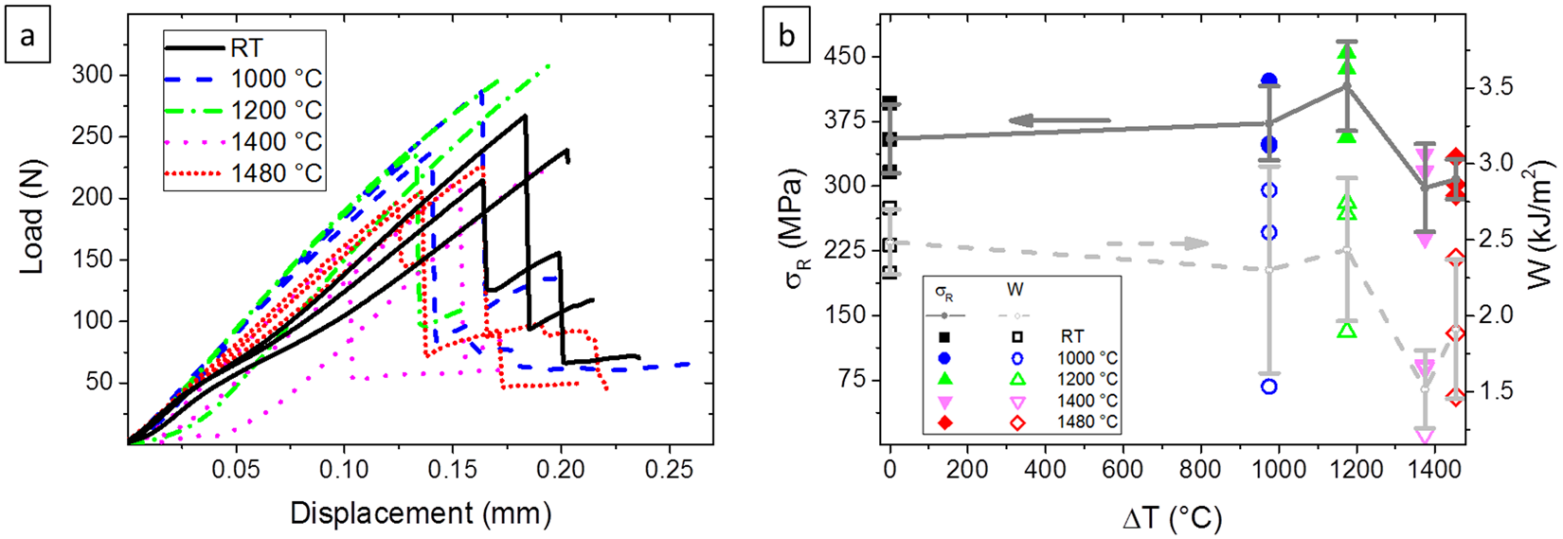
(**a**) Load-displacement curves of 4 pt. for three different bars for each thermal shock. (**b**) Retained flexure strength (σ_R_) vs. thermal shock (ΔT); solid line connects mean σ_R_ values at each ΔT, and calculated work of fracture (W) vs. thermal shock (ΔT); dashed line connects mean W values at each ΔT.

The thermal shock behavior after water-quench is shown in Fig. 6. Both single data and their average value plus dispersion are indicated in the graph. The data of the thermal quenched bars indicate that a thermal shock severity as high as 1500 °C was sustained. The maximum decrease of strength occurring at 1400 °C was 16% of the initial value, and 13% at 1500 °C suggesting that the samples could be shocked at even higher temperature. For the sake of comparison, ZrB_2_-based materials material containing 15 vol. % SiC tested using the water-quenching method, reported critical thermal shock (ΔTC) between 350 °C and 475 °C depending on the matrix composition^29^. For brittle ceramics with poor resistance to thermal shock it can observed that close to the critical thermal temperature, not only the average strength value drops abruptly below 70% of the pristine value, but data dispersion increases dramatically (up to 100%) as reported in refs^29,30^. On the contrary, in the present case, we observed that the data dispersion did not significantly change from RT to thermally shocked samples even at 1500 °C, being around 12%. The low fracture toughness and high elastic modulus are the properties usually penalizing the thermal shock resistance. For these new composites, the damage tolerance of 230 µm (calculated through the Irwin equation: $c=\,Y\frac{{K}_{Ic}^{2}}{\pi {\sigma }_{f}^{2}}$, by assuming that the material contains elliptical flaws in the bulk: $Y=1$) as well as the decrease of modulus could play an effective role in increasing the thermal shock resistance of about three times. Oscillation of the average values, for instance the strength increase after shock at 1200 °C or decrease after shock at 1400 °C can be due to concurrent phenomena such release of residual stresses and oxidation, respectively. Indeed, even if the thermal shock produces new defects, the formation of the cracks is accompanied by residual stress release, which could explain the increase observed after shock at 1200 °C. It is plausible that when the residual tensile stress present into the matrix are released, as consequence of the crack formation (probably smaller than 230 µm), the flexural strength of the matrix increases and reaches the flexural strength of the composite. As it can be seen from the Fig. 6a, the stiffness of the thermal shocked composites up to 1200 °C is constant for the whole test until the flexural failure. In agreement with the lower fracture toughness, obtained at 1500 °C, the work of fracture after the thermal shocks is lower for all the investigated temperatures. However these materials confirm their superior damage tolerant (230 µm at RT and 80 µm at 1500 °C), and ΔTC (higher than 1480 °C) respect to the corresponding ZrB_2_-based bulk ceramics. The obtained values of strength and toughness in function of temperature and after thermal shock confirm the possibility to use these novel UD-UHTCMCs as engineering structural materials for aerospace applications, and provide a useful overview for future developments.

## Conclusions

Novel UHTCMCs based on ZrB_2_ reinforced with 45 vol. % of homogeneously distributed unidirectional carbon fibers were produced. Their mechanical properties in function of temperature and after thermal shock were investigated. Due to the different thermal expansion coefficient between the matrix and the carbon fibers, cracks occur in the matrix and this leads to a flexural strength of about 70 MPa, while the composites fail at 360 MPa as result of the carbon fibers reinforcement. The increase of flexural strength up to 550 MPa at 1500 °C and up to 420 MPa after thermal shock of 1175 °C were attributed to the trade-off between the residual stress release and the formation of new cracks.

## Materials and Methods

### Materials preparation

Commercially available powders were used for the preparation of ceramic composite materials: ZrB_2_ (H.C. Starck, grade B, Germany, specific surface area 1.0 m^2^/g, particle size range 0.5–6 µm, impurities (wt. %): 0.25 C, 2.00 O, 0.25 N, 0.10 Fe, 0.20 Hf), α-SiC (H.C. Starck, Grade UF-25, Germany, specific surface area 23–26 m^2^/g, D_50_ 0.45 µm, Impurities (wt. %): 2.5 O; 0.04 Al, 0.01 Ca; 0.05 Fe; Italian retailer: Metalchimica), Si_3_N_4_ (Baysinid Bayer, Germany, specific surface area 12.2 m^2^/g, D_50_ 0.15 µm, Impurities (wt. %): 1.5 O). Unidirectional fabric ultra-high modulus pitch-derived carbon fibers (TCU312, Supplier: G. Angeloni) were used as carbon preforms. The following composition was prepared:

$$55\,{\rm{v}}{\rm{o}}{\rm{l}}.\,{\rm{ \% }}\,(89{\rm{ \% }}\,{{\rm{Z}}{\rm{r}}{\rm{B}}}_{2}+3{\rm{ \% }}\,{\rm{S}}{\rm{i}}{\rm{C}}+8{\rm{ \% }}\,{{\rm{S}}{\rm{i}}}_{3}{{\rm{N}}}_{4}{)}_{matrix}+45\,{\rm{v}}{\rm{o}}{\rm{l}}.\,{\rm{ \% }}\,{\rm{C}}\,{}_{{\rm{f}}{\rm{i}}{\rm{b}}{\rm{e}}{\rm{r}}{\rm{s}}}$$

where Si_3_N_4_ is added as sintering aid, SiC as reinforcing and oxidation resistant phase.

The ZrB_2_ – SiC - Si_3_N_4_ powder mixture was first prepared by conventional wet ball milling and drying. Aqueous slurries based on this mixture were used to infiltrate UD fabrics that were stacked in 0–0° configuration, according to procedures described in ref.^12^. Hot pressing cycles were then carried out in the range 1800–1900 °C, using a pressure of 30–40 MPa^12^.

The microstructures were analysed on polished and fractured surfaces by field emission scanning electron microscopy (FE-SEM, Carl Zeiss Sigma NTS Gmbh Öberkochen, Germany) and energy dispersive X-ray spectroscopy (EDS, INCA Energy 300, Oxford instruments, UK). X-ray diffraction Bruker D8 Advance apparatus (Bruker, Karlsruhe, Germany).

After sintering, the bulk density was determined by the Archimede method. The relative density, ρ was thus calculated as the ratio of experimental to theoretical value, and the residual porosity deduced as 1 − ρ. Image analysis (Image - Pro Analyser 7.0) was carried out onto SEM micrographs of polished sintered sections to determine the amounts of Cf and porosity.

### Mechanical characterization

4-pt bending strength tests at R.T. were carried out following the standards of advanced technical ceramics. Test bars with dimensions, 25 × 2.5 × 2 mm^3^ (length by width by thickness, respectively) were fractured using a semi-articulated silicon carbide four-point fixture with a lower span of 20 mm and an upper span of 10 mm using a screw-driven load frame (Instron mod. 6025, Instron, High Wycombe, GB) according to EN 843-1 (2004) norm for advanced technical ceramics. With the same machine and fixture we also performed flexural strength tests at 1200 and 1500 °C in Ar flux to limit oxidation effects. For each temperature 3–4 bars were tested, depending on availability. Each test was loaded with a crosshead speed of 1 mm/min.

The fracture toughness (K_Ic_) was evaluated by fracturing chevron notched beams (CNB), following the guidelines of EN 14425-3 (2010) for advanced technical ceramics. The test bars were 25 × 2 × 2.5 mm^3^ (length by width by thickness, respectively) were notched with a 0.1 mm-thick diamond saw; the chevron-notch tip depth and average side length were about 0.12 and 0.80 of the bar thickness, respectively. The specimens were fractured using a fully-articulated steel four-point fixture with a lower span of 20 mm and an upper span of 10 mm using a screw-driven load frame (Instron, 6025). Three specimens were loaded with a crosshead speed of 0.05 mm/min. The “slice model” equation of Munz *et al*.^31^ was used to calculate K_Ic_.

The Young’s modulus was measured using the acoustic method. The input Poisson coefficient values to calculate the major Poisson ratio (ν_12_ = 0.23), and the Young’s modulus along the longitudinal direction (E_1_) were 0.19 and 0.27 for the ZrB_2_ matrix and Cf, respectively^32^. The minor Poisson Ration (ν_21_ = 0.18) and the transverse Young’s modulus (E_2_) were calculated by iterating the acoustic method in order to satisfy the equation: $\frac{{\nu }_{12}}{{E}_{1}}=\frac{{\nu }_{21}}{{E}_{2}}.$ E_1_ was also measured using the resonance frequency method, on bars 2 × 6 × 45 mm.

The thermal shock resistance was determined with the method of the retained strength after water-quenching (20 °C of bath temperature) on 25 mm × 2.5 mm × 2 mm (length by width by thickness, respectively) bars. The experimental critical thermal shock ΔTC was determined following the guidelines outlined by the standard prEN820-3. In particular, ΔTC was identified using a linear interpolation between points that first reduce the average flexure strength of the quenched bars by more than 30% of the mean strength of the as-sintered material. For each quench temperature, at least three specimens were tested.

The thermal expansion coefficient (CTE) was determined up to 1500 °C, with a heating rate of 5 °C/min, under flowing argon, using a dilatometer Netzsch mod. DIL E 402 (Netzsch, Geraetebau, Germany), on 25 mm × 2.5 mm × 2 mm bars.
